# Comparison of voiding vesicoureteral urosonography with fluoroscopic voiding cystourethrography in children with vesicoureteral reflux

**DOI:** 10.12669/pjms.39.4.6665

**Published:** 2023

**Authors:** Bin Yang, Junping Wang, Zhiyan Di, Shasha Zhao, Yali Ma, Yanchao Qu

**Affiliations:** 1 Bin Yang, Department of Urology Surgery, Baoding Children’s Hospital, Baoding 071000, Hebei, China; 2 Junping Wang, Department of Urology Surgery, Baoding Children’s Hospital, Baoding 071000, Hebei, China; 3 Zhiyan Di, Department of Urology Surgery, Baoding Children’s Hospital, Baoding 071000, Hebei, China; 4 Shasha Zhao, Department of Urology Surgery, Baoding Children’s Hospital, Baoding 071000, Hebei, China; 5 Yali Ma, Department of Urology Surgery, Baoding Children’s Hospital, Baoding 071000, Hebei, China; 6 Yanchao Qu, Department of Urology Surgery, Children’s Hospital affiliated to Capital Medical University, Beijing 1000045, China

**Keywords:** Vesicoureteral reflux, Ultrasonography, X-ray, Diagnosis

## Abstract

**Objective:**

To evaluate the value and compliance rate of voiding vesicoureteral urosonography in pediatric vesicoureteral reflux (VUR).

**Methods:**

This is a retrospective study. A total of 80 children with high-risk VUR admitted to Children’s Hospital affiliated to Capital Medical University from December 2018 to December 2020 were selected. All patients underwent voiding urosonography (VUS) and fluoroscopic voiding cystourethrography (VCUG). The sensitivity and compliance of voiding vesicoureteral urosonography were compared, and its application value was evaluated.

**Results:**

A total of 160 PUUs were examined, and all cases were normal. Among them, 56 PUUs had reflux (35.00%, 56/160), 46 PUUs had reflux under both examination methods (28.75%, 46/160), and 10 PUUs were only detected under VUS (6.25%, 10/160). Thirty-four cases of VUR (42.50%, 34/80) were diagnosed by VUS, among which 15 cases were bilateral reflux and 4 cases were unilateral reflux. Twenty-five cases (35.00%, 25/80) were diagnosed by VCUG, among which 10 cases were bilateral regurgitation and five cases were unilateral regurgitation. No significant difference was observed in the detection rate of reflux between the two methods (P=0.432). A total of 146 PUUs were found to be consistent between the two methods (91.25%, 160), including 2 Grade-I reflux, 6 Grade-II reflux, 14 Grade-III reflux, 12 Grade-IV reflux, eight Grade-V reflux, and 104 without reflux, demonstrating SATISFACTORY consistency between the two groups (Kappa=0.885).

**Conclusion:**

Voiding vesicoureteral urosonography has a high coincidence rate in the detection of vesicoureteral reflux in children.

## INTRODUCTION

Urinary tract infection is one of the diseases with high incidence in childhood. Studies have revealed that vesicoureteral reflux (VUR) has a close bearing on the occurrence of renal scarring and upper urinary tract infection. VUR is one of the main causes of urinary tract infection in children.^1^ In particular, some children suffer from recurrent urinary tract infection accompanied by primary VUR.^2^ Although the overall incidence of VUR in children is only about 1%, it accounts for more than a fifth of urinary tract infections. In case of the simultaneous occurrence of the above two conditions, persistent kidney damage and scarring may occur, even pediatric hypertension and chronic kidney damage. To this end, the earlier the diagnosis of VUR is made, the more timely the treatment of recurrent urinary tract infections in children will be.^3^ Imaging examinations are the most preferred examination methods in clinical practice, including ultrasonography, voiding vesicoureterography, and fluoroscopic voiding cystourethrography (VCUG). Conventional ultrasonography, limited by imaging methods, presents poorly represented ureter information. It is basically no longer used in clinical practice, while the latter two methods are still prevalent. VCUG has been considered as the gold standard technique for the diagnosis of VUR.^4^ However, it requires urinary catheterization and fluoroscopic imaging which expose children to radiation. With the rapid development of imaging and related contrast agent technology, ultrasonography has been attached great importance to clinical practice. Several studies have shown VUS to be a valid alternative method for the assessment of VUR.^5^,^6^ While prior studies provided a parallel comparison of VUS and VCUG, data were not obtained in a temporally simultaneous manner. Whether there is consistency between VUR and VCUG needs to be further discussed.^7^

In this paper, 80 children with vesicoureteral reflux who were admitted to our hospital from December 2018 to December 2020 were selected as subjects. The effectiveness of these methods was determined by comparing the application of VUS with VCUG.

## METHODS

This is a retrospective study. A total of 80 children with VUR who were admitted to our hospital from December 2018 to December 2020 and diagnosed with voiding cystourethrography as the gold standard were selected as subjects, with a total of 160 renal ureteral units (PUUs). There were 30 boys aged from one month to 11 years, with an average age of (4.09±0.67) years. There were 50 girls aged from two months to 11 years, with an average age of (4.12±0.81) years.The study was approved by the Institutional Ethics Committee of Children’s Hospital affiliated to Capital Medical University on December 20, 2018 (No.[2018]109), and written informed consent was obtained from all participants.

### Inclusion criteria:

- Children with all examination results on file and complete data;
- Children with high-risk VUR indicated by all indicators;
- Children who voluntarily participated in the study themselves and their families, were aware of all the study contents and signed the informed consent form.

### Exclusion Criteria:

- Children with other severe diseases who cannot move and cannot cooperate with the completion of the examination;
- Children who themselves and their families cannot effectively cooperate with the study;
- Children who cannot undergo examination due to drug contraindications or allergic history.

Color Doppler ultrasound tester (model: Acoustics AIxplorerV), XC6-1 probe, frequency 1-6MHz, CPS real-time ultrasonography software. SonoVue was employed as an ultrasound contrast agent. 5 ml sterile normal saline was injected into the contrast agent and shaken until the lyophilized powder was completely dispersed, so as to prepare phospholipid coated sulfur hexafluoride (SF6) gas microbubbles. Every 3-ml of sterile saline was mixed with 0.2 ml of gaseous microbubbles to form a suspension, and 30-60 ml was prepared for later use. For VCUG contrast agent, two vials of Maglumine Diatrizoate (Manufacture: Shanghai Xudong Haipu Pharmaceutical Co., Ltd.; *Strength:* 20 ml/vial, iodine content 370 mg/ml) were compounded with sterile saline at 1:1 to make 80 ml of meglumine diatrizoate solution for later use.

**Fig.1:**
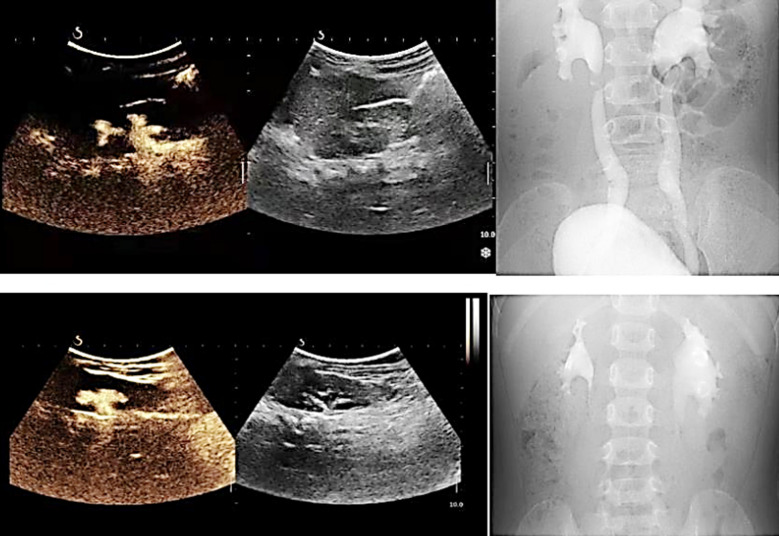
Contrast agent was observed in ureter and renal pelvis on ultrasound and abdominal x-rays respectively.

### VUS method^8:^

Children were placed in supine position, and the abdominal probe (set the probe frequency at 4.5hz) was first applied to have a gray-scale ultrasound for a comprehensive and detailed scan of their kidney, ureter and bladder. Emphasis should be laid on the size of the kidney, the dilatation of the renal pelvis and ureter, and the abnormalities of the dermatomedullary structures. An indwelling catheter was placed and the bladder was emptied. Attached to the tee coupling, the catheter was connected to ultrasound contrast and saline respectively. Normal saline was slowly dripped to the half-filling amount, which was calculated by (age +2)×30mL. Then, a bolus injection of 0.5ml of the prepared contrast microbubble suspension was performed, followed by instillation of normal saline to the filling volume, and the urinary catheter was clamped. After that, the contrast agent was injected via the catheter and converted to CPS mode, with an operating frequency of 1.5 MItz, MI=0.18, and real-time observation for about 5-l0 min. All dynamic images were automatically stored in the instrument. After the examination, all the data were be backed up in a unified manner for timely analysis, and conclusions were drawn.

SonoVue contrast agent was dealt by injection bolus until the microbubbles were evenly distributed in the bladder lumen. Subsequently, the children were guided to urinate, and the enhancement of the bladder, ureter and renal pelvis and calyces were observed in real time. The presence of ultrasound contrast agents in the ureter and renal pelvis indicates VUR. The grade was then determined based on the international VCUG grading criteria, depending on the site of urine reflux in children. VUR is divided into five grades^9^: (1) Grade-I: urinary reflux reaching the lower 1/3 of the ureter; (2) Grade-II: urinary reflux reaching the ureter, renal pelvis, and renal calyx but without dilatation, with normal renal pelvis fornix; (3) Grade-III: mild or moderate dilatation and/or distortion of the ureter, but without/or mild fornix getting blunted; (4) Grade-IV: moderate dilatation and/or distortion of the ureter, with moderate dilatation of renal pelvis, complete disappearance of fornix angle, but most of renal calyx maintaining papillary indentation; (5) Grade-V: severe dilatation and distortion of the ureter, severe dilatation of renal pelvis and renal calyx, and disappearance of the papillary indentation of renal calyx.

**Table-I:** Consistency analysis of the detection of VUS and VCUG.

		VCUG		Total

		VUR(-)	VUR(+)	
VUS	VUR(-)	46	10	56
	VUR(+)	0	104	104
	Total	46	114	160

### VCUG method:

After the catheter is intubated, 60-120 ml of meglumine solution is injected into the catheter. X-ray fluoroscopy machine was employed to observe and determine the distribution of contrast agent in bilateral ureters and renal pelvis, and simultaneously, a full range of photographs were carried out. Note that the urinary catheter needs to be removed during the urination phase observation, and the observation items are the same as in 1.3.3. Reflux is indicated by the presence of contrast in the ureter or renal pelvis. Grading shall also be determined according to the grading criteria listed in 1.3.3.

It should be noted that all children should be followed up at least 24 hour after the relevant examination to observe acute or late allergic reactions. In all of the above analysis of ultrasound images, a team of physicians with at least two years of contrast-enhanced ultrasound (CEUS) experience should be assigned to make the judgment. Usually, the double-blind method is preferred. In case of disagreement, more physicians need to be appointed to make the judgments, and the final result should be decided by voting if necessary.^10^ The accuracy of the results should be ensured.

### Observation items:

(1) Consistency analysis between VUS and VCUG; (2) Comparison of grading results detected by VUS and VCUG.

### Statistical Analysis:

All data in this study were analyzed using statistical software (SPSS 19.00 version). The measurement data was expressed as (*χ̅*±*S*), and the count data as (%). P<0.05 indicates a statistically significant difference. Kappa values were calculated by McNemar test to determine the consistency of test results.

## RESULTS

A total of 160 PUUs were examined, and all cases were normal. Among them, 56 PUUs had reflux (35.00%, 56/160), 46 PUUs had reflux under both examination methods (28.75%, 46/160), and 10 PUUs were only detected under VUS (6.25%, 10/160). Thirty-four cases of VUR (42.50%, 34/80) were diagnosed by VUS, among which 15 cases were bilateral reflux and four cases were unilateral reflux. Twenty-five cases (35.00%, 25/80) were diagnosed by VCUG, among which 10 cases were bilateral regurgitation and five cases were unilateral regurgitation. No significant difference was observed in the detection rate of reflux between the two methods, with no statistically significant difference (P=0.432).

**Table-II:** Comparison of grading results detected by VUS and VCUG

VCUG								

		No reflux	I	II	III	IV	V	Total
VUS	No reflux	104	0	0	0	0	0	104
	I	3	2	0	0	0	0	5
	II	3	0	6	0	0	0	9
	III	2	0	1	14	0	0	17
	IV	1	0	0	1	12	0	14
	V	1	0	0	1	1	8	11
	Total	114	2	7	16	13	8	160

A total of 146 PUUs were found to be consistent between the two methods (91.25%, 160), including two Grade-I reflux, six Grade-II reflux, 14 Grade-III reflux, 12 Grade-IV reflux, eight Grade-V reflux, and 104 without reflux, demonstrating SATISFACTORY consistency between the two groups (Kappa=0.885).

## DISCUSSION

Vesicoureteral reflux (VUR) may give rise to repeated urinary tract infections in children to a certain extent, given the close correlation between the two.^11^,^12^ If no timely diagnosis is made, the normal growth and development of children may be affected.^13^ Currently, diagnostic techniques for VUR have been continuously explored and improved.^14^ Considering the particularity of the patient population, higher requirements are put forward for the safety and accuracy of detection.^15^ Fluoroscopic voiding cystourethrography (VCUG), which is widely used at present, exerts its remarkable effect via digital film and pulse technology. However, repeated examination using VCUG will cause harm to the health of children because of its significant radioactivity.^16^ In contrast, voiding urosonography (VUS) stands out among many contrast methods by virtue of its non-radioactivity.^17^ VUS has been proven to be effective in diagnosis and more sensitive than VCUG. When the latter is used for examination, the urinary tract is photographed intermittently and discontinuously, so the process of urethral filling and excretion cannot be observed in real time and carefully, resulting in certain omissions. However, due to the large limitations of VUS in the display of the posterior urethra, the operator is required to pay attention to the method during the operation so as to observe the urethral structure more clearly.^18^ In consideration of the growing concern that X-rays may harm children’s health, the CEUS+ function of the AIxplorer V model produced by SuperSonic Imagine was used to conduct VUS examinations. CEUS+ has been accepted by an increasing number of patients and their families for its features of radiation-free, long duration of observation, high sensitivity, specificity, and low cost. The CEUS+ function of the AIxplorer V model (SuperSonic Imagine), a full-information contrast agent imaging (CCI) technology based on the full-channel stack platform, provides more nonlinear microbubble signals. With its characteristics of high frame rate and high resolution, the stability of microbubbles can be guaranteed to the maximum extent without being easily destroyed, and its display time can be guaranteed for better continuous dynamic observation of lesions as well as continuous and effective case analysis. It also boasts excellent temporal and spatial resolution, clearer image hierarchies and preferable observation of detailed lesions.

It can be known that a total of 160 PUUs were examined by analyzing the results of this study. No significant difference was observed in accuracy between the two methods, and a higher detection rate of reflux was found in the examination performed by VUS. VUS has a high coincidence rate in the detection of VUR in children. It boasts of reducing adverse effects such as radiation and has appreciable clinical application value. According to Kim et al^19^, considering the similar performance of detecting and grading VUR, better detection ratio of intrarenal reflux, and advantage of being a radiationfree modality, VUS may have potential to be the initial modality of choice for evaluating VUR in children with urinary tract infections. Mane et al^20^ pointed out that VUS is a good imaging modality when compared to VCUG to assess pediatric VUR, in view of its superior diagnostic performance, feasibility and radiation safety for children.

Nonetheless, certain limitations remain visible in the application of voiding vesicoureteral urosonography. For example, in the process of examination, it is impossible to check all target organ information at the same time and in the same section, so it is necessary to set the examination sequence and section in advance in order to obtain more information^21^. Especially when patients have abnormal bilateral ureteral reflux, it is easy to miss on the other side. In practical application, we should pay more attention to the consistency of application and make a decision in time.

### Limitations of this study:

The number of subjects included in this study was limited, so the conclusions drawn may not be very convincing. In addition, this study was a retrospective study with limited data integrity and homogeneity. It is necessary to further design a randomized controlled trial to verify the conclusions of this study.

## CONCLUSION

Voiding vesicoureteral urosonography has a high coincidence rate in the detection of vesicoureteral reflux in children. It boasts of reducing adverse effects such as radiation and has high clinical application value.

### Authors’ Contributions:

**BY and** **YQ** designed this study, prepared this manuscript, are responsible and accountable for the accuracy and integrity of the work.

J**W and ZD** collected and analyzed clinical data.

**SZ and** **YM** Data analysis**.** significantly revised this manuscript.
